# Persistence of pulmonary tertiary lymphoid tissues and anti-nuclear antibodies following cessation of cigarette smoke exposure

**DOI:** 10.1186/1465-9921-15-49

**Published:** 2014-04-22

**Authors:** Mathieu C Morissette, Brian N Jobse, Danya Thayaparan, Jake K Nikota, Pamela Shen, Nancy Renée Labiris, Roland Kolbeck, Parameswaran Nair, Alison A Humbles, Martin R Stämpfli

**Affiliations:** 1 Department of Pathology and Molecular Medicine, McMaster Immunology Research Centre, McMaster University, Hamilton, ON, Canada; 2 Honours Molecular Biology & Genetics Co-op Program, McMaster University, Hamilton, ON, Canada; 3 Medical Sciences Graduate Program, McMaster University, Hamilton, ON, Canada; 4 Department of Medicine, Firestone Institute of Respiratory Health at St. Joseph's Healthcare, McMaster University, Hamilton, ON, Canada; 5 MedImmune LLC, Gaithersburg, MD, USA; 6 MDCL 4011, 1280 Main Street West, Hamilton, Ontario L8S 4K1, Canada

**Keywords:** Tertiary lymphoid tissue, Autoantibodies, Autoimmunity, COPD, Experimental model, Inflammation

## Abstract

Formation of pulmonary tertiary immune structures is a characteristic feature of advanced COPD. In the current study, we investigated the mechanisms of tertiary lymphoid tissue (TLT) formation in the lungs of cigarette smoke-exposed mice. We found that cigarette smoke exposure led to TLT formation that persisted following smoking cessation. TLTs consisted predominantly of IgM positive B cells, while plasma cells in close proximity to TLTs expressed IgM, IgG, and IgA. The presence of TLT formation was associated with anti-nuclear autoantibody (ANA) production that also persisted following smoking cessation. ANAs were observed in the lungs, but not the circulation of cigarette smoke-exposed mice. Similarly, we observed ANA in the sputum of COPD patients where levels correlated with disease severity and were refractory to steroid treatment. Both ANA production and TLT formation were dependent on interleukin-1 receptor 1 (IL-1R1) expression. Contrary to TLT and ANA, lung neutrophilia resolved following smoking cessation. These data suggest a differential regulation of innate and B cell-related immune inflammatory processes associated with cigarette smoke exposure. Moreover, our study further emphasizes the importance of interleukin-1 (IL-1) signaling pathways in cigarette smoke-related pulmonary pathogenesis.

## Introduction

Cigarette smoking is well know for its adverse health impacts, being a leading risk factor for most cancers, as well as cardiovascular and respiratory diseases [1]. Currently, emphasis has been placed on reducing smoking prevalence; however, a greater understanding of the mechanisms of cigarette smoke’s adverse effects on human health is equally relevant given the addictive nature and chronic persistence of cigarette smoking, and the burden tobacco use places on healthcare [1].

It is widely accepted that inflammatory processes elicited by cigarette smoke play an important role in the pathogenesis of chronic obstructive pulmonary disease (COPD). Over the past decades, great advances have been made in our understanding of molecular pathways involved in driving cigarette smoke-induced inflammation [2,3]. Emphasis has been placed on innate immune cells, including alveolar macrophages and neutrophils, for their destructive potential as reactive oxygen species and proteases released by these cells may cause tissue damage [4]. More recently, there is emerging interest in the role of the adaptive immune system [5]. It has been postulated that persistent inflammation associated with cigarette smoking may promote autoimmune processes [5], as evidenced by the occurrence of circulating autoantibodies in COPD patients [6-8]. Mechanisms driving these autoimmune processes remain poorly understood. Of particular interest is the formation of pulmonary tertiary lymphoid tissues as a potential inductive site of autoantibodies in patients with advanced COPD.

Cytokines play a key role in orchestrating cigarette smoke-induced inflammatory processes. To date, more than 50 cytokines and chemokines of interest have been reported [9]. Of particular interest is interleukin-1 (IL-1) as it regulates innate and adaptive immune processes. Doz and colleagues demonstrated a critical role for interleukin-1 receptor 1 (IL-1R1), the receptor for both IL-1α and IL-1β, in cigarette smoke-induced neutrophilia [10]. Parallel studies by Churg *et al.* showed that IL-1R1 KO mice were protected against cigarette smoke-induced emphysema formation [11]. More recently, we reported that IL-1R1 signaling pathways were required for dendritic cell expansion and T cell activation following cigarette smoke exposure [12]. The relative importance of IL-1R1 in tertiary lymphoid tissue (TLT) formation and autoantibody production is currently unknown.

The objective of this study was to investigate whether cigarette smoke exposure leads to the formation of pulmonary TLT and autoantibody production using a pre-clinical model of cigarette smoke exposure, as well as to determine the importance of IL-1R1 in these processes. We report the formation of TLT in mice exposed to cigarette smoke that persists following smoking cessation. We further show the presence of broad-spectrum autoantibodies recognizing anti-nuclear antigens in the lungs that persist following smoking cessation. ANA were also observed in the sputum of COPD patients. Studies in gene deficient mice showed that TLT and ANA formation were IL-1R1-dependent. Our study shows that chronic cigarette smoke exposure induces adaptive immune processes that persist following smoking cessation. These findings further emphasize the importance of IL-1 signaling pathways in cigarette smoke-related pulmonary pathologies as well as B cell and innate immune responses.

## Methods

### Animals

Female BALB/c mice (6-8 weeks old) were purchased from Charles River Laboratories (Montreal, PQ, Canada). Female, 6-8 weeks old C57BL/6 and IL-1R1^-/-^ (C57BL/6 background) mice were purchased from The Jackson Laboratories (Bar Harbor, ME). All mice were kept in a 12-h light-dark cycle with food and water *ad libidum*. The McMaster University Animal Research Ethics Board approved all experiments described in this study.

### Cigarette smoke exposure

Mice were exposed to cigarette smoke for 4 days, 8 weeks and 24 weeks using a whole-body exposure system (SIU48, PROMECH LAB AB, Vintrie, Sweden) as previously described in detail [13,14]. Briefly, mice were exposed to the mainstream smoke of twelve 3R4F reference cigarettes (University of Kentucky, Lexington, USA) with filters removed. Mice were exposed 5 days per week, twice daily, for 50 minutes/exposure. Total particulate matter in the exposure chamber was measured weekly and ranged from 600 to 700 μg/L. We previously reported that cigarette smoke exposure is well tolerated and results in cotinine and carboxyhemoglobin levels comparable to human smokers [13]. Control animals were exposed to room air only.

### Collection of mouse specimens

Bronchoalveolar lavage (BAL), lung tissue, and blood were collected as previously described [13,14]. Total cell counts in the BAL were determined using a haemocytometer. BAL cytospins were prepared for differential cell counts and stained with Hema 3 as per the manufacturer’s instructions (Biochemical Sciences Inc., Swedesboro, New Jersey, USA). At least 300 leukocytes were counted per cytospin. BAL fluid was stored at -80°C. Blood was collected by retro-orbital bleeding, serum was obtained and stored at -80°C. For histological assessment, the left lung was inflated with 10% neutral buffered formalin at a constant pressure of 30 cm of water, and then fixed in 10% neutral buffered formalin for 72 hours.

### Sputum samples

All clinical samples were collected with patients’ informed written consent and ethical approval from the Research Ethic Board at McMaster University. Sputum induction was performed as described by Pizzichini *et al.*[15]. Spirometry was performed according to standards of the American Thoracic Society.

### CXCL13 and anti-nuclear antibodies measurement

Bronchoalveolar lavage levels of CXCL13 were measured by ELISA according to manufacturer’s specifications (R&D systems, Minneapolis, MN). Anti-nuclear antibodies were measured by ELISA. Nuclei were isolated from TC-1 cells (mouse lung epithelial cell line; grown according to ATCC guidelines) and A549 cells (human adenocarcinoma cell line; grown according to ATCC guidelines) according to manufacturer’s specifications (Nuclear extraction kit, Millipore, CA). NUNC 96-well plates (Nalge Nunc international, NY) were dry-coated with 1 μg of nuclear protein per well in 100 μl of water overnight at 37°C. Wells were washed three times (0.05% TWEEN-20 in PBS) prior to incubation with either mouse BALF (1:2 dilution), mouse serum (1:500 dilution) or human sputum supernatant (1:10 dilution) at room temperature (RT) for two hours. Plates were washed 3 times (0.05% TWEEN-20 in PBS) prior to incubation with the detection antibody for 1 hour at RT. Total mouse ANA were detected using rabbit anti-mouse conjugated to horseradish peroxidase (HRP) (1:1000; Invitrogen, NY). For isotype specific detection, wells were incubated with biotinylated goat anti-mouse IgM, IgA, or IgG (1/1000; Sigma Aldrich), washed, and incubated with streptavidin-HRP. Total human ANA were detected using anti-human immunoglobulin conjugated to HRP (1:1000; Invitrogen, NY). Wells were washed then incubated with chromogen TMB substrate (BioFX Laboratories, MD) and optical density was measured at 450 nm. The assay was validated using the serum and BALF of the autoimmune New Zealand Black (NZB) mouse as a positive control (Additional file 1: Figure S1).

### Histological measurements

Formalin-fixed, paraffin-embedded lungs were cut into 4 μm thick cross-sections and stained with hematoxylin and eosin. Four photos at a 16x magnification were taken for every lung. Whole lung cross-section area (Lung_area_ [pixel^2^/10^10^]) as well as the number of bronchus-associated TLT (TLT_number_) were determined using the Image J Software and identified according to their distinctive morphology. Data are expressed as bronchus-associated TLTs per lung area (TLT_number_/Lung_area_).

### Immunohistochemical stainings

Three micrometer thick sections from formalin-fixed, paraffin-embedded lungs were stained by immunochemistry for B220, CD138, IgM, IgA and IgG protein expression. Briefly, lung sections were deparaffinized in xylene and rehydrated in ethanol:water. Endogenous peroxidases were blocked in 3% H_2_O_2_ in methanol. Citrate buffer antigen retrieval was performed (45 minutes). Sections were blocked with 1% swine serum in TBS 0.01% Tween 20. Sections were stained at 4°C overnight with either rat anti-mouse B220 (1:50; Abcam), rabbit anti-mouse CD138, biotinylated goat anti-mouse IgM, IgA or IgG. Antibodies were detected with a biotinylated goat anti-rat IgG (B220) or anti-rabbit IgG (CD138) (1:100; 1 h RT; BioLegend) followed by streptavidin-HRP (DACO) or directly by streptavidin-HRP (IgM, IgA, IgG). Staining was visualized using 3-amino-9-ethylcarbazole (AEC) substrate reagent. Sections were counterstained with hematoxylin. Negative staining controls are presented in Additional file 1: Figure S2.

### Statistical analysis

Statistical analyses were performed using StatView Software. Two-group comparisons were made using an unpaired Student’s t-test with a significant threshold at 0.05. Experimental protocols with more than two groups were compared using a two-way ANOVA (significant threshold at 0.05) followed, if applicable, by a Bonnferoni post-hoc test.

## Results

### Chronic cigarette smoke exposure leads to persistent tertiary lymphoid tissue formation in the lung

**Figure 1:**
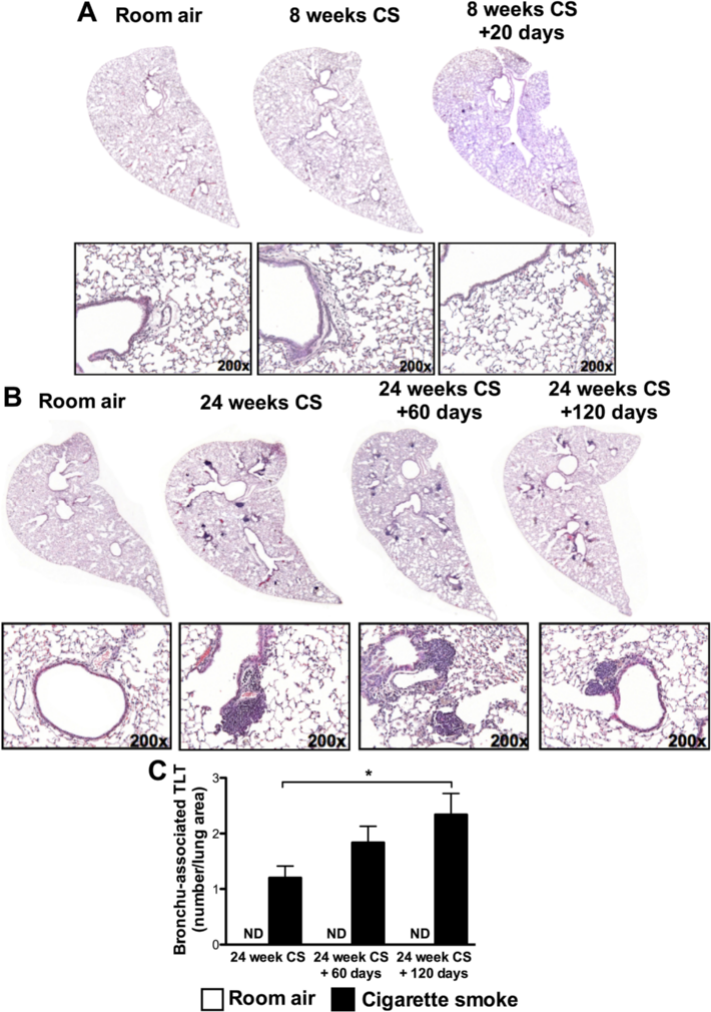
**Chronic cigarette smoke exposure leads to pulmonary tertiary lymphoid tissue (TLT) formation that persists following smoking cessation.** BALB/c mice were exposed to room air (open bars) or cigarette smoke (solid bars) for **(A)** 8 weeks ± 20 days of smoking cessation or for **(B)** 24 weeks ± 60 and 120 days of smoking cessation. Lung sections were stained with hematoxylin and eosin (H&E). **(C)** Pulmonary TLT were quantified using Image J software. Data represent means ± SEM; n = 5 per group. *p < 0.05.

A characteristic histopathological feature of advanced COPD is the emergence of pulmonary tertiary lymphoid tissue (TLT) [16]. Using a murine model, we investigated the time course of cigarette smoke-induced TLT formation and whether TLTs persist following smoking cessation. BALB/c mice were exposed to cigarette smoke for 4 days, 8 weeks, and 24 weeks. Control groups were exposed to room air only. No TLTs were observed after 4 days (data not shown) and 8 weeks of cigarette smoke exposure (Figure 1A). In contrast, TLTs were present in the lungs of mice exposed to cigarette smoke for 24 weeks (Figure 1B). No TLT formation was observed in age-matched room air-exposed animals, providing evidence that formation of these lymphoid structures was a consequence of cigarette smoke exposure rather than aging. Similarly, we observed TLT formation in C57BL/6 mice following 24, but not 8, weeks of cigarette smoke exposure (data not shown). Of note, TLT persisted in the lungs following smoking cessation for 60 and 120 days (Figure 1B). Quantification of TLT provided evidence of their expansion following smoking cessation (Figure 1C). These data demonstrate that cigarette smoke exposure leads to the formation of TLTs that persist following smoking cessation.

### Characterization of B cells within pulmonary tertiary lymphoid tissue and localization of immunoglobulin-secreting cells

**Figure 2:**
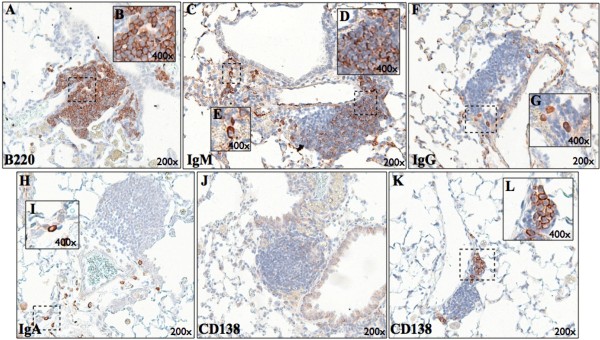
**Plasma cells are found in close proximity with pulmonary tertiary lymphoid tissues (TLT) that mainly contain naïve B cells.** BALB/c mice were exposed to cigarette smoke for 24 weeks and lung sections were stained by immunochemistry for **(A)** B cells (B220), **(C)** IgM, **(F)** IgG, **(H)** IgA and **(J-K)** plasma cells (CD138). **(B)** Tight clusters of B220 expressing B cells. **(D)** Cells expressing membrane-bound IgM within a pulmonary TLT. **(E)** IgM-, **(G)** IgG- and **(I)** IgA-secreting cell (cytoplasmic staining) found adjacent to pulmonary TLTs. **(J)** Absence of plasma cells inside pulmonary TLT which are generally found near pulmonary TLT or **(K-L)** vessels.

Cell types found in cigarette smoke-induced pulmonary TLTs include T cells, B cells, dendritic cells, and macrophages [16]. Using immunohistochemistry, we characterized the phenotype of B cells in TLTs of BALB/c mice exposed to cigarette smoke for 24 weeks. Cells within TLTs stained positive for B220, a commonly used B cell marker (Figure 2A and 2B). The predominant isotype expressed within TLTs was IgM (Figure 2C and 2D), while only few IgG^+^ (Figure 2F) and IgA^+^ (Figure 2H) cells were observed. TLT B cells displayed a distinct membrane-staining pattern for IgM (Figure 2D) and lacked expression of the plasma cell marker CD138^+^ (Figure 2J). Of note, IgM^+^ (Figure 2E), IgG^+^ (Figure 2G), and IgA^+^ (Figure 2I) cells, displaying a distinct cytoplasmic staining pattern were observed in areas immediately adjacent to TLTs, often nearby blood vessels. This staining pattern is indicative of plasma cells, as B cell that differentiate into antibody producing plasma cells loose surface expression of membrane bound immunoglobulin while greatly expanding their immunoglobulin synthesis machinery. This gives plasma cells a characteristic cytoplasmic immunoglobulin-staining pattern. This observation was supported by the presence of cells expressing the plasma cell marker CD138^+^ in similar locations (Figure 2K and 2L).

### Cigarette smoke exposure elicits local but not systemic production of anti-nuclear autoantibodies

**Figure 3:**
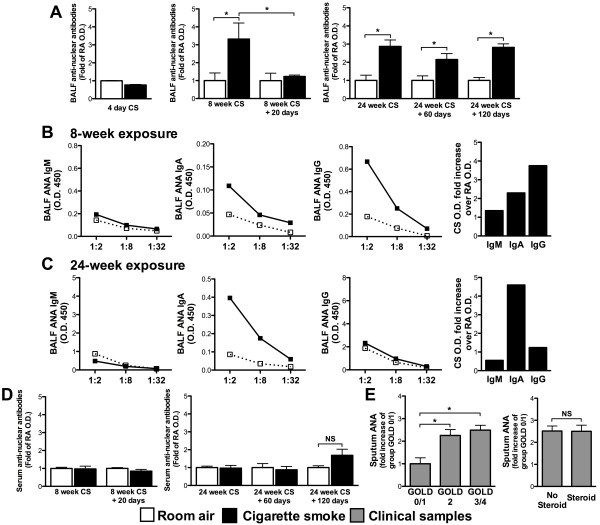
**Cigarette smoke exposure elicits pulmonary but not systemic anti-nuclear antibodies production.** BALB/c mice were exposed to room air (open bars) or cigarette smoke (solid bars) for 4 days, 8 weeks ± 20 days of smoking cessation or for 24 weeks ± 60 and 120 days of smoking cessation. ANA levels were measured by ELISA in the **(A)** bronchoalveolar lavage fluid (BALF) and antibody isotypes dissected in mice exposed for **(B)** 8 weeks and **(C)** 24 weeks. **(D)** ANA levels were also measured in the serum of mice exposed for 8 weeks ± 20 days of smoking cessation or for 24 weeks ± 60 and 120 days of smoking cessation. **(E)** ANA levels were measured in the sputum of different GOLD stage subjects (see Tables 1 and 2 for complete description) and expressed as fold increase of GOLD 0/1 subjects. **(A and D)** Data represent means ± SEM; n = 5-10 mice per group. *p < 0.05. **(B and C)** Data represent the values obtained from pooled samples from each group. Fold changes were calculated by using the average O.D. value of the corresponding room air-exposed group as baseline expression (fold increase of 1).

Emerging evidence suggests that cigarette smoking elicits autoantibody responses in susceptible individuals [6-8]. We next investigated whether formation of TLT and accumulation of plasma cells in the lungs of cigarette smoke-exposed mice was associated with autoantibody production. We chose to investigate the presence of broad-spectrum autoantibodies against nuclear antigens; a type of autoantibody widely associated with autoimmune disorders. Anti-nuclear antibodies (ANA) were assessed in the BALF (local) and the serum (systemic) of cigarette smoke and room air-exposed mice. While no increase in ANA was observed following 4 days of cigarette smoke exposure, exposure to cigarette smoke for 8 and 24 weeks was associated with significantly increased levels of ANA in the BALF (Figure 3A). No increase in BALF ANA levels was observed in room air-exposed mice. In mice exposed to cigarette smoke for 8 weeks, increased ANA levels returned to baseline following smoking cessation for 20 days. In contrast, ANA remained elevated for more than 120 days post-cessation in mice exposed to cigarette smoke for 24 weeks (Figure 3A). Further analysis of BALF ANA following 8 weeks of cigarette smoke exposure revealed that the main isotypes expressed were IgG and IgA (Figure 3B). After 24 weeks of smoke exposure, the predominant ANA isotype in the BALF was IgA (Figure 3C), an isotype adapted for mucosal secretion. Expression of ANA was specific to the lungs, as no increase in ANA was observed in the serum of cigarette smoke-exposed mice (Figure 3D). This suggests that following 8 weeks of cigarette smoke exposure, and in the absence of TLT, pulmonary ANA expression is transient and resolves following smoking cessation. In contrast, following chronic exposure to cigarette smoke, and in the presence of TLT, pulmonary ANA persist following smoking cessation.

### ANA levels are elevated in COPD patients and are refractory to steroid treatment

Previous clinical studies focused on serum analysis of autoantibodies [7,17,18]. To investigate whether cigarette smoking was associated with accumulation of autoantibodies in the human lung, as observed in our animal model (Figure 3A), ANA were assessed in the sputum of COPD patients. ANA levels were significantly elevated in the sputum of GOLD stage 2/3/4 COPD patients compared to GOLD stage 0/1 (Figure 3E) (see Table 1 for patient descriptions). Moreover, steroid-based therapy had no effect on sputum ANA levels (see Table 2 for patient descriptions) (Figure 3E). To our knowledge, this is the first study to report that autoantibodies accumulate locally in the sputum of COPD patients and that they are refractory to steroid-based therapy.

**Table 1:** Patient characteristics

**Variables**	**Gold 0/1 (n = 7)**	**Gold 2 (n = 23)**	**Gold 3/4 (n = 17)**
**Age, yr**	**55.7 (9.7)**	**62.3 (7.9)**	**61.0 (8.7)**
**Gender, F/M**	**3/4**	**11/12**	**8/9**
**FEV**_**1**_**, % pred.**	85.4 (5.8)^*^	**59.2 (9.2)**^**‡**^	**36.2 (8.2)**^**#**^
**FEV**_**1**_**/FVC, %**	73.0 (11.3)^*^	**55.0 (9.9)**^**‡**^	**38.3 (8.9)**^**#**^

Data are expressed as mean(SD).

Values with different superscript symbols are significantly different from each other.

**Table 2:** Patient characteristics

**Variables**	**Gold 2/3/4 no steroid use (n = 10)**	**Gold 2/3/4 Steroid use (n = 19)**
**Age, yr**	66.8 (6.5)^*^	**59.3 (8.4)**^**‡**^
**Gender, F/M**	**4/6**	**10/9**
**FEV**_**1**_**, % pred.**	57.0 (12.0)^*^	**45.4 (13.3)**^**‡**^
**FEV**_**1**_**/FVC, %**	54.9 (12.8)^*^	**44.1 (9.1)**^**‡**^

Data are expressed as mean(SD).

Values with different superscript symbols are significantly different from each other.

### Neutrophilic inflammation Resolves following chronic cigarette smoke exposure

**Figure 4:**
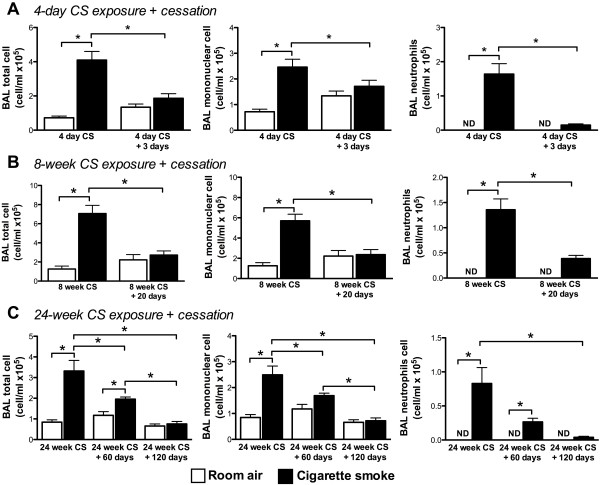
**Resolution of lung inflammation is delayed following chronic cigarette smoke exposure.** BALB/c mice were exposed to room air (open bars) or cigarette smoke (solid bars) for **(A)** 4 days ± 3 days of smoking cessation, **(B)** 8 weeks ± 20 days of smoking cessation or for **(C)** 24 weeks ± 60 and 120 days of smoking cessation. Data show total cell number, mononuclear cells and neutrophils in the BAL. Bars represent means ± SEM; n = 5 per group. *p < 0.05.

In COPD patients, pulmonary inflammation may persist following smoking cessation. We therefore investigated whether the presence of TLT and the elevated ANA levels were associated with persistent inflammation following smoking cessation. BALB/c mice were exposed to cigarette smoke for 4 days, 8 weeks, and 24 weeks, and the BAL cellular profile was assessed 18 hours after the last smoke exposure and following smoking cessation. We observed significantly increased total cell numbers (TCN), mononuclear cells (MNC), and neutrophils following 4 days, 8 weeks, and 24 weeks of cigarette smoke exposure (Figure 4A-C). In mice exposed to cigarette smoke for 4 days (Figure 4A), increased numbers of total cells, MNC and neutrophils resolved within 3 days of smoking cessation. In mice exposed for 8 weeks (Figure 4B), TCN and MNC returned to baseline within 20 days, while the neutrophilia persisted. In mice exposed to cigarette smoke for 24 weeks (Figure 4C), TCN, MNC, and neutrophils declined but remained significantly elevated compared to room air controls following smoking cessation for 60 days. Following 120 days of smoking cessation, TCN and MNC returned to baseline levels, while neutrophilia resolved by >95%. These findings suggest that the cellular inflammatory profile in the BAL resolves following smoking cessation; although resolution of inflammation appears to be delayed following prolonged cigarette smoke exposure.

### Role of IL-1 signaling in cigarette smoke-induced neutrophilia, elevated pulmonary anti-nuclear antibodies and TLT formation

**Figure 5:**
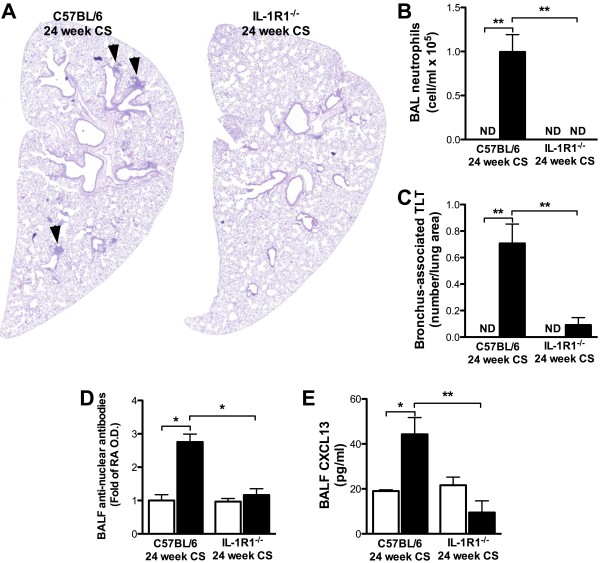
**Neutrophil recruitment, pulmonary tertiary lymphoid tissue development and increased ANA levels are dependent on IL-1R1 expression.** C57BL/6 and IL-/R1^-/-^ mice were exposed to room air (open bars) or cigarette smoke (solid bars) for 24 weeks. **(A)** Lung sections were stained with hematoxylin and eosin (H&E). **(B)** Total neutrophils number was assessed in the BAL. **(C)** Pulmonary TLT were quantified using Image J software. **(D)** Pulmonary ANA and **E)** CXCL13 levels were measured in the BALF by ELISA. Data represent means ± SEM; n = 5 per group. *p < 0.05, **p < 0.01.

IL-1R1 signaling pathways play a critical role in cigarette smoke-induced neutrophilia, dendritic cell accumulation, and T cell activation [10,12]. We therefore investigated the role of IL-1R1 signaling in the generation of TLT and ANA following chronic cigarette smoke exposure. Confirming our previous observations, no neutrophils were found in the BAL of IL-1R1^-/-^ mice following 24 weeks of cigarette smoke exposure (Figure 5B). Moreover, neither TLT nor elevated ANA were observed in the lungs of cigarette smoke-exposed IL-1R1-deficient mice (Figure 5A,C,D). CXCL13, a B cell-attracting chemokine that has recently been implicated in cigarette smoke-induced TLT formation [19], was significantly increased in cigarette smoke compared to room air-exposed wild type mice. In contrast, levels of CXCL13 were not increased in cigarette smoke-exposed IL-1R1^-/-^ mice (Figure 5E). These findings suggest that IL-1R1 signaling pathways play a central role in cigarette smoke-induced innate immune inflammatory processes as well as processes associated with adaptive immune responses such as the formation of tertiary lymphoid tissues and autoantibody production.

## Discussion

Clinical evidence suggests that cigarette smoking leads to TLT formation in the lung; however, the mechanisms that lead to the formation of these tertiary lymphoid tissues and their association with autoimmune processes are at present unknown. The objective of the current study was to investigate the mechanisms of TLT formation and autoantibody production using a mouse model of cigarette smoke exposure.

To investigate cigarette smoke’s impact on TLT formation, we utilized a well-characterized murine model of cigarette smoke exposure [13,14,20]. We previously reported that cigarette smoke exposure is well tolerated and leads to cotinine and carboxyhemoglobin levels comparable to human smokers thus validating the experimental approach [13]. In the current study, we report that exposure to cigarette smoke led to pulmonary TLT formation that persisted following smoking cessation. B cells within TLTs preferentially expressed membrane-bound IgM, while plasma cells expressing IgM, IgG, or IgA were located in areas adjacent to TLTs. These findings suggest that TLTs are a site of B cell activation, leading to plasma cell differentiation and antibody production within the lungs.

A key observation of this study is that formation of lung tertiary lymphoid tissues was strongly associated with persistently increased levels of ANA following smoking cessation. We analyzed anti-nuclear antibodies (ANA) since they are a broad-spectrum class of autoantibodies that are associated with a number of autoimmune disorders in both humans and experimental models [21,22]. The increase in ANA levels was observed in the lungs, but not in the circulation of cigarette smoke-exposed mice. While we observed a transient increase in ANA IgG following 8 weeks of cigarette smoke exposure, ANA IgA was the predominant isotype after 24 weeks. This shift towards IgA provides further evidence of local autoantibody production, as IgA is the isotype associated with mucosal tissues. This may signify that these autoimmune processes are confined to the lungs and lack the systemic component that is observed in other autoimmune diseases such as systemic lupus erythematosus, rheumatoid arthritis, or Sjögren’s syndrome [21,23,24].

While lung TLT are a characteristic feature of advanced COPD [16], the function of this lymphoid structures remain poorly understood. Studies by Bracke and colleagues [19] suggest that TLTs do not contribute to emphysema formation in cigarette smoke-exposed mice. While autoantibodies were present in cigarette smoke exposed mice prior to TLT formation, sustained presence of ANA after smoking cessation was only observed in the presence of pulmonary TLTs. It is plausible that these structures might be a site of continuous activation of B cells even following smoking cessation and the generation of autoantibody-producing plasma cells.

In line with our observations in cigarette smoke-exposed mice, we show a significant increase in ANA levels in the sputum of GOLD stage 2/3/4 COPD patients compared to GOLD stage 0/1 subjects thus providing evidence that sputum autoantibodies levels correlate with disease severity. Of note, ANA levels in the sputum were steroid insensitive; similar levels were observed in patients that received steroid treatment compared to patients that were not treated with steroids. While there is increasing evidence that autoantibodies are detected in the circulation of COPD patients [9-11], several reports did not confirm these observations [19,20]. Our findings suggest that autoantibodies associated with COPD may be more reliably detected in sputum and provide evidence that cigarette smoke elicits autoimmune processes specifically within the lungs.

Mechanistically, we demonstrated that cigarette smoke-induced TLT formation and ANA production is IL-1R1-dependent. These findings further highlight the importance of IL-1R1 signaling pathways in cigarette smoke-induced inflammation. We, and others, have previously shown that cigarette smoke-induced neutrophilia [10,25,26] and formation of emphysematous damage is, at least in part, IL-1R1 dependent [11]. Of note, IL-1R1 signaling pathways were required for the induction of CXCL-13, a chemokine that plays a critical role in TLT formation following cigarette smoke exposure [19], suggesting that the absence of TLT in the lung of IL-1R1-/- mice may be caused by a lack of CXCL13 upregulation. Of particular interest to our current study is that dendritic cell expansion and T cell activation following cigarette smoke exposure are also IL-1R1 dependent [12]. Hence, the ability of cigarette smoke to induce autoimmune processes may be subsequent to dendritic cell activation as postulated by Cosio and colleagues [5].

To the best of our knowledge, we are the first to document that IL-1R1 plays a critical role in cigarette smoke-induced ANA and TLT formation. While little is known about the role of IL-1R1 in autoimmunity, Croker *et al.* reported that autoimmune processes observed in *Ptp6n*^-/-^ mice (deficient in SHP1 protein) were IL-1R1 dependent [27]. Interestingly, autoimmunity in this model was likely triggered by the host’s microflora, as germ-free *Ptp6n*^-/-^ mice did not exhibit the phenotype. While mechanisms leading to inflammation and autoimmunity remain to be elucidated in the *Ptp6n*^-/-^ mice, the underlying processes are similar to the IL-1R1 dependency of cigarette smoke-induced ANA production and BALT formation.

**Figure 6:**
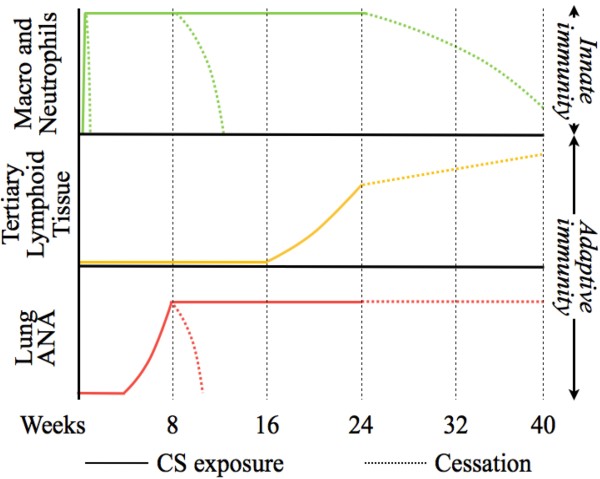
**Kinetic of the innate and adaptive responses induced by cigarette smoke exposure and the impact of smoking cessation.** Resolution of the innate immune response (macrophages and neutrophils) measured in the BAL is markedly delayed when preceded by chronic exposure but can happen. Features of adaptive immune response (pulmonary TLT formation and elevated ANA) requires chronic exposure to be initiated but does not resolve following cessation. Moreover, the delay in the resolution of the innate immune response is associated with the activation of the adaptive immune response.

Contrary to the sustained production of ANA and persistence of TLT, neutrophilia resolved following smoking cessation. Of interest, the time course of resolution was a function of the duration of cigarette smoke exposure. Neutrophilia resolved within 3 days in an acute exposure protocol (4 days). Following 24 weeks of cigarette smoke exposure, inflammation resolved more slowly, as significantly increased neutrophil numbers were observed 60 days following smoking cessation, ultimately approaching baseline only after 120 days of cessation. This relates to what is observed clinically where inflammation may persist for a prolonged period following smoking cessation [28,29]. It is currently not understood whether ANA contribute to this delayed resolution. Immune complexes are well known to propagate neutrophilic inflammation [30] and promote neutrophil and monocyte survival *in vitro*[31,32]. Alternatively, it is plausible that ANA attenuate cigarette smoke-induced inflammatory processes via opsonizing cellular debris and improving their clearance by phagocytic cells. Moreover, whether autoantibodies perpetuate or attenuate pathogenic mechanisms may depend on antibody specificity and isotype, as well as, the microenvironment within the target tissue. While the consequences of elevated ANA in the lungs of cigarette smoke-exposed mice are currently unknown, it is of interest that adaptive immune processes such as autoantibody production and TLT formation persisted following smoking cessation, while innate inflammatory processes resolved (Figure 6).

Cigarette smoke exposure is a leading risk factor for many diseases such as cancer, cardiovascular diseases, and COPD which reflects the complexity of its effects on the human body. Data presented here demonstrate that cigarette smoke induces local ANA production that is associated with the presence of pulmonary TLTs. Our data further highlights the importance of IL-1 signaling pathways in innate and adaptive immune inflammatory processes associated with COPD pathology.

## Competing interests

The authors have read the journal's policy and have the following conflicts: RK and AAH are full time employees of MedImmune, a wholly owned subsidiary of AstraZeneca. This does not alter the authors' adherence to all the *Respiratory Research* policies on sharing data and materials.

## Authors’ contributions

MCM was responsible for conceptualization of mouse experiments, experimentation, data analysis, and preparation of the manuscript. BNJ, JKN, DT, and PS provided support for mouse experimentation, discussion, and manuscript preparation. RNL and RK assisted discussion of data and provided feedback for the manuscript. PN provided clinical samples and provided input on experimental design and data interpretation. AAH assisted in conceptualization of experiments, discussion of data, and provided feedback for the manuscript. MRS supervised the project and played an instrumental part in conceptualizing experiments and the preparation of the manuscript. All authors read and approved the final manuscript.

## Supplementary Material

Additional file 1 Online supplement. Click here for file
