# Supplementary material for: Persistence of pulmonary tertiary lymphoid tissues and anti-nuclear antibodies following cessation of cigarette smoke exposure
Source: Respir Res. 2014 Apr 22;15(1):49. doi: 10.1186/1465-9921-15-49 (PMC4021094; doi:10.1186/1465-9921-15-49)
Supplement: Additional file 1 — Online supplement. [file 1465-9921-15-49-S1.doc]

ONLINE SUPPLEMENT

**Persistence of pulmonary tertiary lymphoid tissues and anti-nuclear antibodies following cessation of cigarette smoke exposure**

Mathieu C. Morissette, Brian N. Jobse, Danya Thayaparan, Jake K. Nikota, Pamela Shen, Renée N. Labiris, Roland Kolbeck, Parameswaran Nair, Alison A. Humbles, Martin R. Stämpfli


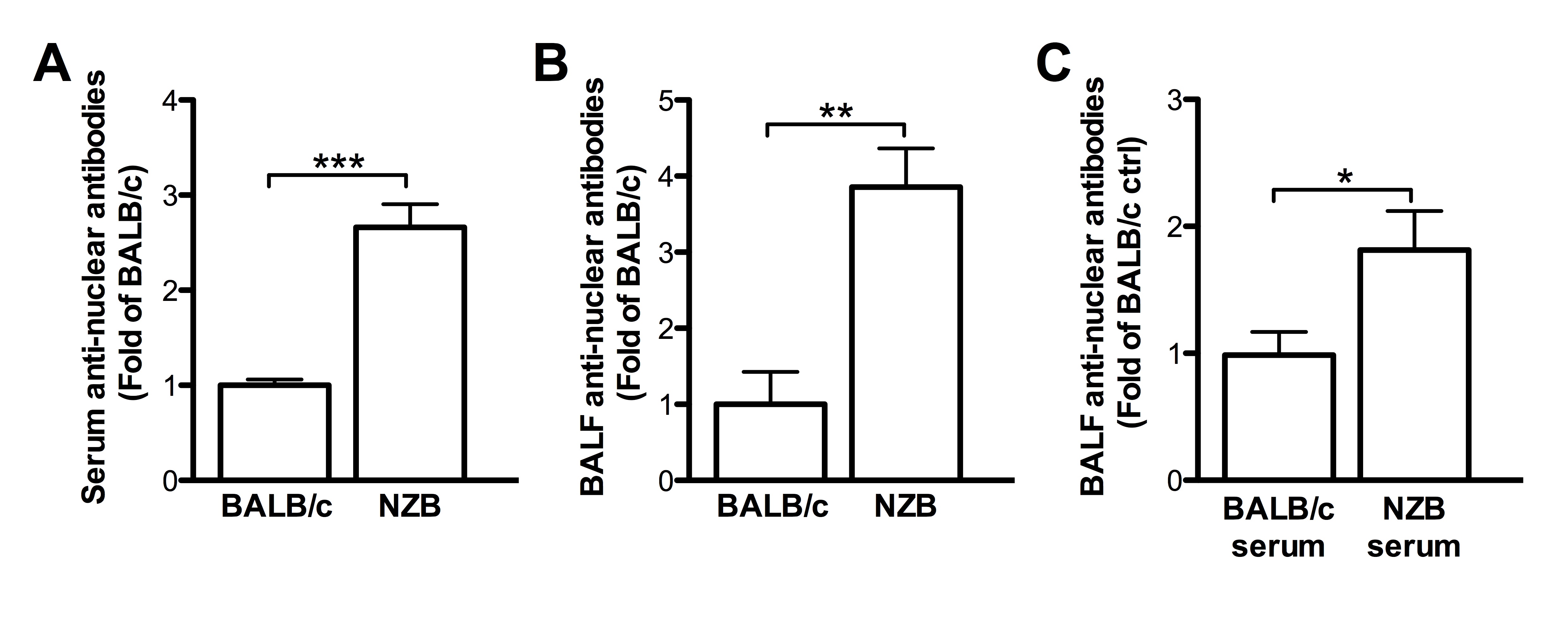


***Figure S1.*** *Effectiveness of the ELISA against anti-nuclear antibodies (ANA) in detecting ANAs in the serum and lung of the autoimmune New Zealand Black (NZB) mouse.* The ANA ELISA was used to assess the levels of ANA in **A**) the serum and **B**) the bronchoalveolar lavage fluid (BALF) of BALB/c mice and, as a positive control, the autoimmune prone NZB mouse. **C**) Serum from BALB/c mice or NZB mice was injected intraperitonealy (i.p.) in BALB/c mice. ANA levels were measure after one week in the BALF and significantly increase in the mice that received NZB serum i.p..


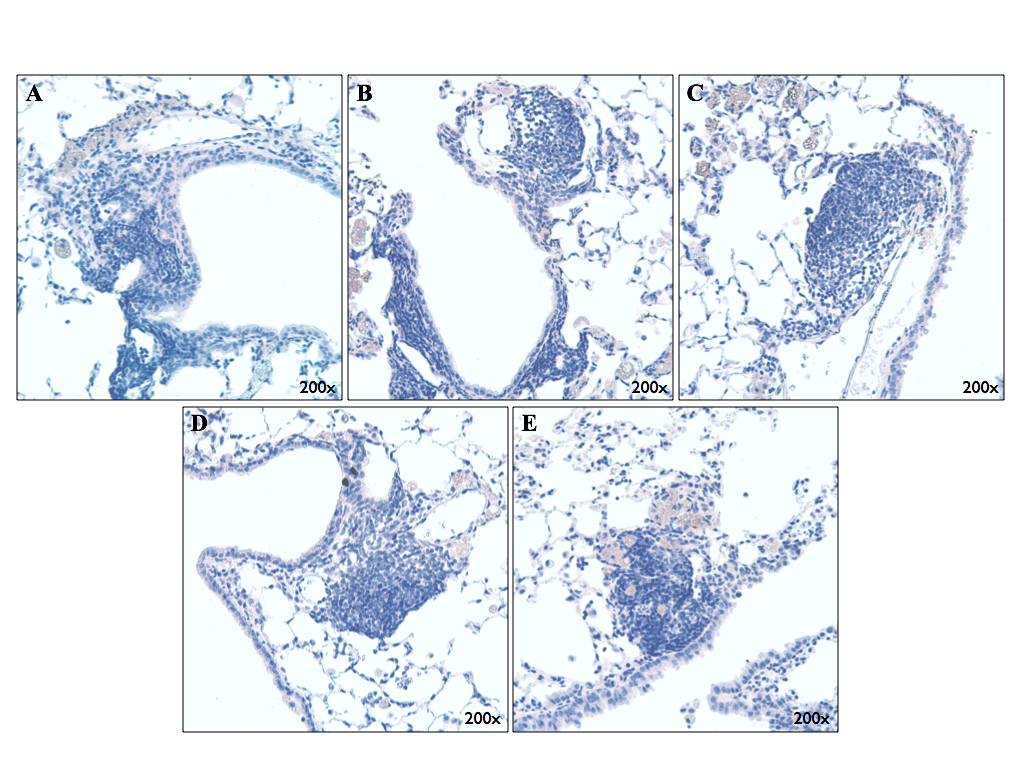


***Figure S2.*** *Negative controls for the immunohistochemical stainings.* Sections were treated as detailed in the method section up to the blocking step. Prior to substrate exposure and counter stain, tissue sections were treated with **A)** no primary, no secondary, and no detection antibodies, **B)** streptavidin-HRP only, **C)** biotinylated anti-rat followed by streptavidin-HRP, **D)** biotinylated anti-rabbit followed by streptavidin-HRP or **E)** biotinylated anti-goat followed by streptavidin-HRP.
